# Using eye movements to detect visual field loss: a pragmatic assessment using simulated scotoma

**DOI:** 10.1038/s41598-020-66196-2

**Published:** 2020-06-17

**Authors:** Daniel S. Asfaw, Pete R. Jones, Laura A. Edwards, Nicholas D. Smith, David P. Crabb

**Affiliations:** Division of Optometry and Visual Sciences, School of Health Sciences, City, University of London, London, EC1V 0HB UK

**Keywords:** Human behaviour, Biomarkers, Computational science

## Abstract

Glaucoma is a leading cause of irreversible sight-loss and has been shown to affect natural eye-movements. These changes may provide a cheap and easy-to-obtain biomarker for improving disease detection. Here, we investigated whether these changes are large enough to be clinically useful. We used a gaze-contingent simulated visual field (VF) loss paradigm, in which participants experienced a variable magnitude of simulated VF loss based on longitudinal data from a real glaucoma patient (thereby controlling for other variables, such as age and general health). Fifty-five young participants with healthy vision were asked to view two short videos and three pictures, either with: (1) no VF loss, (2) moderate VF loss, or (3) advanced VF loss. Eye-movements were recorded using a remote eye tracker. Key eye-movement parameters were computed, including saccade amplitude, the spread of saccade endpoints (bivariate contour ellipse area), location of saccade landing positions, and similarity of fixations locations among participants (quantified using kernel density estimation). The simulated VF loss caused some statistically significant effects in the eye movement parameters. Yet, these effects were not capable of consistently identifying simulated VF loss, despite it being of a magnitude likely easily detectable by standard automated perimetry.

## Introduction

Glaucoma is a chronic eye disease affecting 1 in 28 people aged 40–80 years^1^. It is characterised by progressive, irreversible visual field (VF) loss. Early detection is therefore crucial^2^. Currently, however, as many as 50% of cases remain undiagnosed^3–5^, or are diagnosed late: after substantial vision loss has already occurred^6^. At present, successful detection of glaucoma requires a detailed assessment by a specialist clinician, including measurements of intraocular pressure (IOP), VF loss by standard automated perimetry, and inspection of the optic nerve head. Unfortunately, many adults do not attend routine eye-checks due to associated costs (real and perceived), lack of awareness, aversion to the methods used, and lack of understanding about their purpose^7,8^.

One way to improve glaucoma detection would be to perform proactive screening. However, this is not economical using traditional technologies, owing to the high cost of the personnel and equipment involved^9^. What is needed, therefore, is an inexpensive, automated screening tool to identify high-risk individuals. Modern eye tracking technologies may be able to provide such a solution.

Previous studies have shown that natural eye movements are altered in instances of glaucomatous VF loss. For example, our research has demonstrated that glaucoma patients exhibit differences in saccade frequency and gaze spread when free-viewing images, relative to normally sighted controls^10^. More recently, we found similar differences in eye-movements between the two eyes of glaucoma patients with asymmetric VF loss, when free-viewing pictures monocularly^11^. Several other studies have also likewise confirmed a link between *simulated* VF loss and altered eye movements^12–18^.

The idea of using natural eye movements for disease detection is exciting, as eye movements can be recorded easily and cheaply using ordinary commercial technologies (hardware that is already becoming increasingly ubiquitous in people’s homes, via computer screens, TVs, and smartphones)^19,20^. Furthermore, unlike conventional VF assessments, which require sustained concentration during a protracted test that many patients find demanding^21–23^, natural eye movements require no explicit task, and could even be monitored at home while individuals go about their everyday activities (e.g., watching TV). In short, natural eye movements appear attractive as a cheap and easy way of screening for glaucoma^11,24,25^.

However, despite existing evidence that VF loss alters eye movements, it remains unclear whether these changes are sufficiently great to be *clinically useful*. Exactly what level of sensitivity and specificity a test requires to be clinically useful is difficult to define. However, standard automated perimetry, a well-established tool for detecting VF loss, is able to discriminate between healthy vision and moderate-or-advanced VF loss with almost 100% accuracy. For example, Cello *et al*. reported that an automated perimeter (FDT C-20) displayed 100% sensitivity and 100% specificity for detecting advanced VF loss (mean defect (MD) between –12 and –22 dB), and 96% sensitivity and 96% specificity for moderate VF loss (MD between −6 and −12 dB)^26^. Similarly, Budenz *et al*. reported 100% sensitivity and 96% specificity for detecting moderate or advanced defects using the Humphrey Field Analyzer (Swedish interactive threshold algorithm (SITA) and SITA Fast algorithms)^27^.

In the present study, we used simulated VF loss to explore the power of natural eye movements to detect eye disease. Simulation of VF loss allowed systematic manipulation of key VF loss characteristics, such as location and size, to quickly collect large sets of data in a relatively short period of time, and to control for individual variability by recruiting a relatively homogenous cohort of healthy, young adults^28^.

Several techniques have been developed previously to simulate VF loss. One approach is to use contact lenses with a region of opacity^29–31^. However, this method results in a poorly localised VF loss and causes an unrealistic dimming across the retina^32^. Recent studies have therefore focused instead on using eye tracking to control the position of an artificial VF loss on a computer, relative to the current point of fixation (gaze-contingent presentation)^13,28,33^. We have recently reported one such gaze-contingent VF loss simulator (“GazeSS”), capable of applying real patient’s VF defects (measured by standard automated perimetry) onto dynamic film content^33,34^.

The purpose of the present work was to consider whether natural eye movements can provide a clinically useful biomarker for glaucomatous VF loss. We contended that, to be clinically useful, eye movements should be at least be robust enough to identify substantial VF loss consistently when all of the key confounding variables are controlled (e.g., the environment, exact form of VF loss, and participants’ age and general health). To assess this, we asked normally sighted young adults to watch videos and images with or without a gaze-contingent simulated VF loss (based on clinical data from a single glaucoma patient). Unlike most previous studies, our primary interest was not to establish whether any changes in eye movements were statistically significant. Instead, we used the receiver operating characteristic (ROC) to examine the diagnostic accuracy (i.e. sensitivity, specificity) of natural eye movements in discriminating healthy eyes from those with surrogates of glaucomatous VF loss. We investigated individual eye movement features and their combinations, including saccade amplitude, proportion of saccades landing on the pre-VF loss (SLV) location, spread of saccade endpoints (measured using bivariate contour ellipse area; BCEA), and consistency of fixation locations in time between participants (using kernel density estimation; KDE). Further details on how these variables were computed are given in the *Methods* section.

## Results

### Statistical analysis results

**Figure 1:**
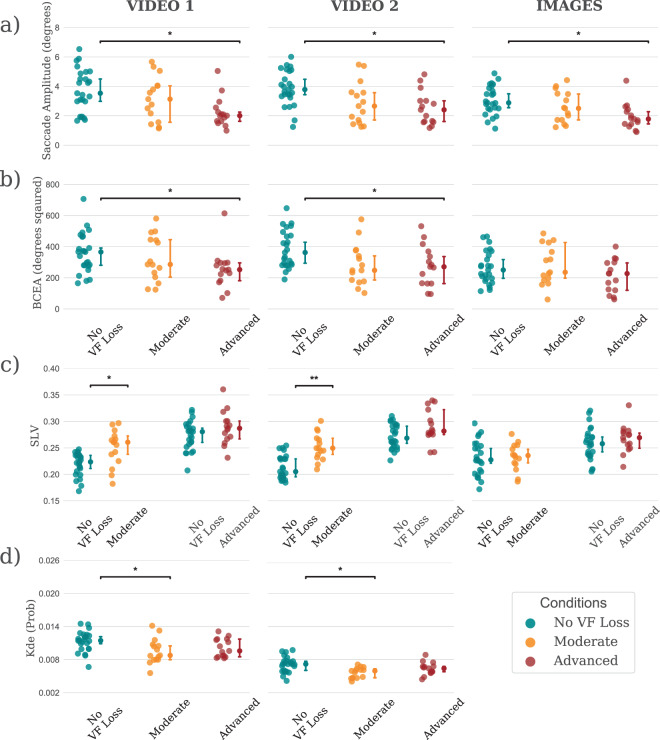
Median values (±95% confidence intervals) for (**a**) median saccade amplitude, (**b**) bivariate contour ellipse area (BCEA), (**c**) proportion of saccades landing on the pre-scotoma locations (SLV), and (**d**) kernel density estimation (KDE) probabilities, split into experimental conditions (no VF loss, moderate, and advanced) and stimulus type (video 1, video 2, and images). Statistically significant differences are denoted with an asterisk (* for p < 0.05 and ** for p < 0.001).

Figure 1 shows the median (±95% confidence intervals; CI) for each of four eye movement parameters (saccade amplitude, bivariate contour ellipse area, saccades landing on the pre-VF loss locations, and kernel density estimation probability), each measured for three visual impairment conditions (no VF loss, moderate VF loss, and advanced VF loss). See *Methods* for definitions of each of these four measures and details of how they were computed.

The specific results from each measurement are described in detail below. However, the overall pattern revealed several statistically significant differences, but indicated that no single parameter provided a consistent, unambiguous biomarker for detecting VF loss. See Table S1 (Supplemental Material) for  results of the statistical analysis. Note that for the analyses reported here and in Table S1, eye movement data from the three images were combined (concatenated) into a single stream, for ease of analysis and reporting. Analysis of individual eye movements from each image showed that there was no statistically significant difference on eye movements within each group (for example in terms of saccade amplitude: F(2, 48) = 2.5, p = 0.92 for no VF loss group; F(2, 28) = 3.22, p = 0.05 for moderate VF loss group and F(2, 28) = 0.75, p = 0.45 for advanced VF loss group).

### Saccade amplitude

In terms of saccade amplitude (Fig. 1a), a Kruskal-Wallis H test showed a statistically significant main effect of visual impairment condition for all stimuli (for video 1: χ^2^(2) = 8.67, *p* = 0.013; for video 2: χ^2^(2) = 7.87, *p* = 0.019; and for images: χ^2^(2) = 10.2, *p* = 0.006). Pairwise comparisons using Mann–Whitney *U* tests (Bonferroni corrected for three comparisons) revealed that the median saccade amplitude in the advanced VF loss group was significantly reduced in video 1 (U = 79, *p* = 0.003, η^2^ = 0.23), video 2 (U = 76, *p* = 0.003, *η*^2^ = 0.24), and images (U = 68, *p* = 0.001, *η*^2^ = 0.28), compared to the no VF loss group. However, there were no statistically significant differences between no VF loss and moderate VF loss, or between moderate and advanced VF loss (both *p* > 0.05).

A follow-up analysis of saccade amplitudes as a function of time (using linear regression) indicated that average saccade amplitude decrease over time when viewing images (example in image 1 (Figure S1), t(57) = −2.36, *p* = 0.02 for No VF loss group; t(57) = −2.63, *p* = 0.01 for moderate VF loss group; and t(57) = −2.76, *p* = 0.008 for advanced VF loss group) but not when watching video 1 (*p* > 0.05) and video 2 (*p* > 0.05). There was no statistically significant difference in the rate at which median saccade amplitude varied with time between no VF loss and the VF loss groups (*p* > 0.05).

### Bivariate contour ellipse area (BCEA)

In terms of BCEA (Fig. 1b; distribution of saccade endpoints), a Kruskal-Wallis H test showed a statistically significant main effect of visual impairment condition for all stimuli (for video 1: χ^2^(2) = 16.1, *p* < 0.001; for video 2: χ^2^(2) = 18.9, *p* < 0.001; and for images: χ^2^(2) = 6.6, *p* = 0.03). Pairwise comparisons using Mann–Whitney *U* tests (Bonferroni corrected for three comparisons) revealed that the average BCEA for advanced VF loss group was significantly reduced compared in video 1 (U = 94, *p* = 0.010, *η*^2^ = 0.17) and video 2 (U = 100, *p* = 0.020, *η*^2^ = 0.15), compared to the no VF loss group. However, there were no statistically significant differences between no VF loss and moderate VF loss, or between moderate and advanced VF loss (both *p* > 0.05). With the three images, Mann–Whitney *U* tests (Bonferroni corrected for three comparisons) found no statistically significant difference between the no and moderate VF loss (U = 193, *p* = 0.99, *η*^2^ = 0.004) or between the no and advanced VF loss groups (U = 143, *p* = 0.330, *η*^2^ =  0.040).

### Saccades landing on the pre-VF loss locations (SLV)

The proportion of saccade endpoints landing on pre-saccade VF loss locations in video 1 (*U* = 81, *p* = 0.002, *η*^2^ = 0.22) and video 2 (*U* = 52, *p* < 0.001, *η*^2^ = 0.36) was higher in the moderate VF loss group compared to the no VF loss group (Fig. 1c). However, there was no statistically significant difference between no VF loss and advanced VF loss (p > 0.05).

### Kernel density estimate (KDE) probability

Kruskal-Wallis H tests found a statistically significant difference in the mean KDE scores (Fig. 1d) when viewing video 1 (χ^2^(2) = 11.4, *p* = 0.003) and video 2 (χ^2^(2) = 9.1, *p* = 0.01). Mann–Whitney *U* tests (Bonferroni corrected for three comparisons) revealed that the average KDE probability score of the moderate VF loss group in video 1 (U = 72, *p* = 0.002, *η*^2^ = 0.26) and video 2 (U = 91, *p* = 0.01, *η*^2^ = 0.18) was significantly reduced compared to the no VF loss group.

### Receiver operating characteristic (ROC) analysis results

**Figure 2:**
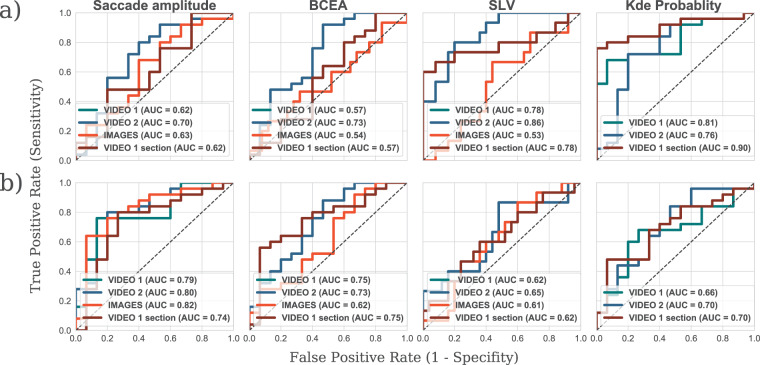
ROC curves and the AUC scores showing the separation between (**a**) the no VF loss and moderate (**b**) the no VF loss and advanced simulated VF losses for the measures described in Fig. 1. The separation was best between no and moderate VF loss in terms of the SLV and KDE probability score, and between no and advanced VF loss in terms of saccade amplitude and BCEA.

The results from the preceding analyses revealed multiple statistically significant differences between no VF loss and moderate VF loss in terms of KDE probability, and between no VF loss and advanced VF loss in terms of saccade amplitude and BCEA. To assess whether these statistical differences are sufficient to robustly discriminate between individuals with/without VF loss, we computed the Receiver Operator Characteristics (ROCs) shown in Fig. 2.

There was a modest separation between the moderate and no VF loss groups  (Fig. 2a) in terms of BCEA for video 2 (area under the curve (AUC) = 0.72) and KDE probability for video 1 (AUC = 0.76), and likewise between the no and advanced VF loss groups (Fig. 2b) in terms of saccade amplitude for video 1 (AUC = 0.78), and video 2 (AUC = 0.79), and images (AUC = 0.83). Overall, our results indicate that modest separation between the no VF loss and moderate groups could be achieved using KDE probability (76% sensitivity and 80% specificity). Similarly, modest separation could be achieved between no VF loss and advanced VF loss using saccade amplitude (80% sensitivity and 80% specificity).

### Improving separation performance

**Figure 3:**
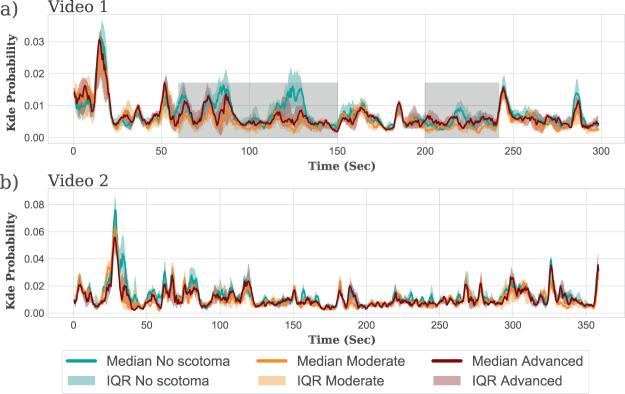
KDE analysis of fixation data, including KDE probability scores as a function of time for (**a**) video 1 and (**b**) video 2. The solid lines show the median values for each group. The shaded regions indicate interquartile range. The average (median) probability score for the participants in the moderate group was smaller than for the no VF loss group (p < 0.05). This difference was more substantial in the grey shaded region of video 1.

To see whether we could further improve separation between groups, we performed two additional analyses. First, we examined the temporal trace from KDE probability analysis (Fig. 3) and identified two sections of video 1 (totaling ~120 sec) where the separation between the no and moderate VF loss groups appeared most pronounced (grey shaded regions in Fig. 3). These regions were selected based on visual inspection of the temporal trace in Fig. 3 (minimal overlap between the two medians ± IQR values), and the results reported below did not qualitatively differ if the exact periods were made slightly longer or shorter. In terms of content, these sections of the video appeared particularly monotonous, with no highly salient objects, e.g. moving vehicles or important landmarks. During these periods participants in no VF loss group tended to ‘rest’ at the centre of the screen, whereas participants with moderate VF loss exhibited more active ‘search’ strategies. Using data from only these cherry picked subsections improved discrimination performance from AUC_all_ = 0.81 to AUC_subsection_ = 0.90. Pairwise comparison between the groups showed the difference between the moderate and no VF loss group was statistically significant (Mann-Whitney *U* test; U = 65, p = 0.002, Bonferroni adjusted for three comparisons) in the grey shaded region of video 1 (Fig. 3).

**Figure 4:**
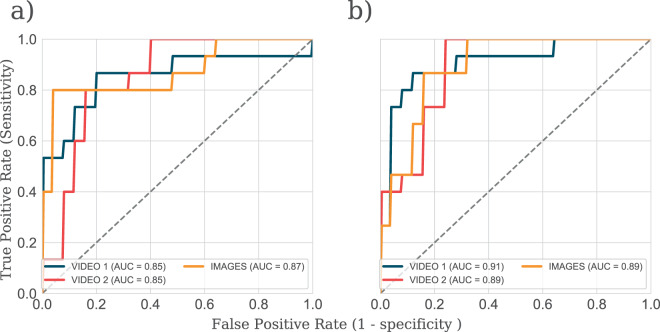
ROC after analysing multiple eye movement parameters to separate (**a**) no VF loss and moderate VF loss group, (**b**) no VF loss and advanced VF loss.

In a second attempt to improve discrimination performance, we extracted multiple saccadic features and analysed using a machine learning technique (for technical details, see *Methods: Further analysis to improve separation performance*). A leave-one-out technique was used to estimate the CI of classification sensitivity and specificity. Using these features improved the separation between no VF loss and moderate VF loss to AUC = 0.85 for video 1, 0.85 for video 2, and 0.87 for images (Fig. 4a). Similarly, the separation between no VF loss and advanced VF loss increased to AUC = 0.91 for video 1, 0.89 for video 2 and 0.89 for images (Fig. 4b). Average sensitivity for correctly identifying a moderate VF loss at a fixed specificity of 90% was 80% for video 1; 60% for video 2 and 60% for images. Similarly, the average sensitivity for correctly identifying advanced VF loss at a fixed specificity of 90% was 88% for video 1; 48% for video 2 and 48% for images. The individual ROCs from three stimuli exhibited very similar AUC scores. In short, these results indicated that using multiple eye movement parameters can improve VF loss detection. However, the performance was still modest relative to the current ‘gold standard’ ophthalmic measures (see Introduction).

## Discussion

This study assessed the sensitivity and specificity of natural eye movements as biomarkers for glaucomatous VF loss. Consistent with previous studies^10,11^, our data showed that natural eye movements are altered by VF loss when viewing videos and images, and the observed differences were statistically significant. Participants with simulated advanced VF loss exhibited smaller saccade sizes and a reduction in the spread of saccade endpoints (Bivariate Contour Ellipse Area; BCEA) compared to participants with no VF loss. A novel measure based on kernel density estimation (KDE) also differed significantly between conditions. However, ROC analysis revealed that these effects were relatively small, despite the substantial levels of VF loss simulated. Using any single measure, our data showed a modest separation between the no and moderate VF loss groups (AUC = 0.81), and between the no and advanced VF loss groups (AUC = 0.82).

To improve separation, we further attempted examining eye movements only in the most informative parts of the videos, and tried combining eye movement metrics to make an ensemble discrimination. These approaches resulted in encouraging performance gains (AUC ≈ 0.9), and indicate that with further development (i.e., better hardware and data analysis techniques), natural eye movements may have the potential to provide a useful biomarker for gross screening of VF loss. However, a discriminative ability of 0.9 still remains relatively poor by clinical standards, given that even the smallest VF defects that we simulated would be detected by automated perimetry with near 100% sensitivity and specificity^27^.

Part of the reason for this low discriminability may be the fact that eye-movements were recorded binocularly. Detecting VF loss from a binocular assessment will always be limited by the fact that binocular vision is often better preserved than vision in either eye alone (even when considering the better seeing eye^35^). It is likely that greater accuracy could be achieved by making the assessment more similar to conventional perimetry: with the use of monocular viewing (patching), head-restraints, fixations targets, and simple stimuli. This, however, we feel would be a mistake. The principle attraction of using natural eye-movements is the ease and accessibility by which data can be acquired. These characteristics would be lost if participants were forced to comply with a rigorous testing protocol. It may be that this in turn places a hard limit on the sensitivity and specificity of the approach. However, this may be acceptable if natural eye-movements are viewed as complementary to traditional diagnostic tools, such as perimetry and structural examinations, and not as a like-for-like replacement. Furthermore, even at this stage natural eye-movements could potentially be useful for assessing hard to reach patient groups, such as those who lack the physical or cognitive capacity to undergo conventional perimetry.

Our data are in general agreement with existing findings. Previous studies have shown that gaze-contingent peripheral VF loss leads to shorter saccade amplitudes when observers perform visual search tasks^16,17,36^ or view pictures freely^37^. Real glaucoma patients have likewise been found to exhibit reduced saccade amplitudes when watching hazard perception videos^38^ or freely viewing pictures^11^, although conflicting results have also been reported in this regard^39,40^. Glaucoma patients also exhibit a smaller spread of fixations (measured using BCEA)^38,41^ and smaller saccade amplitudes^10,11^ when viewing pictures freely.

Several study limitations should be acknowledged. First, the system used to simulate the peripheral VF loss (GazeSS)^33^ operates by applying gaze-contingent blur to regions of the VF. Real patients, however, often report additional symptoms, such as glare and spatial distortions^42,43^. Thus, the simulation does not entirely accurately mimic the effects of binocular VF perfectly, and it would be instructive to replicate our results with real patients in future. Furthermore, the simulated VF loss was based on data from a single patient with a VF defect near the fovea. Since VF loss can be highly heterogeneous across patients, different forms of VF loss (in terms of severity and location) may alter eye movements differently. Therefore, it would be instructive to systematically assess how eye movements differ as a function of VF loss distribution or location and this could be the subject of future work. It is also important to note that participants in the present study experienced an extremely acute onset of simulated vision loss. This is in contrast to real visual impairment, which often progresses gradually over many years^44^.

In terms of improving the discriminative power of eye movements, future work should focus on determining the principles that make a stimulus more or less informative by investigating a variety of video clips that can elicit different eye movements. For example, our pilot study involving ten people indicated that short clips of sports scenes (football, gymnastics and cycling) were less discriminatory. Future studies should also investigate methods of combining eye movement metrics with other easily obtainable data sources (e.g., interocular pressure, demographics, family history), and focus on obtaining larger datasets that could be used to train ‘deep learning’ networks that may be capable of identifying more subtle signs of eye disease^45,46^. Furthermore, better approaches that consider the effect of centre bias on natural eye movements could be explored to help provide better separation between the study groups. For example, using techniques proposed by Marsman, *et al*.^47^ to identify scenes with high viewing priorities prior to computing the KDE. Future work could also consider other techniques that have described characteristics of attention and eye movement changes during the viewing of pictures.

In summary, the present study demonstrated that participants with simulated glaucomatous VF loss exhibited smaller saccades, explored the screen less, and fixated at different locations than the participants with no visual impairment. However, despite these differences being statistically significant, sensitivity and specificity analyses showed that eye movements alone were only able to provide modest separation between participants with/without substantial VF loss. Further development is required before eye movements can provide a clinically useful biomarker for eye-disease in practice.

## Methods

### Task overview

The previously described GazeSS software^33^ was used to simulate two binocular VF conditions^35,48^ (“moderate loss”, and “advanced loss”). Both conditions were based on perimetric data from a single patient with an established diagnosis of glaucoma (measurements taken longitudinally, using standard automated perimetry). Young adults with healthy vision were asked to watch short video clips and images, either with no simulated VF loss, or under one of the two simulated field loss conditions (between-subjects design). We analysed eye movements using common eye movement parameters as well as using a novel measure designed to index the extent to which the observers with simulated VF loss looked at the same screen locations as the no VF loss (control) group.

### Participants

Fifty-five young adults (mean age: 24 years, SD: 5) with normal vision were recruited via advertisements placed in the vicinity of the City, University of London. Normal vision was defined as having no history of eye disease and good performance on the functional vision tests described below. Three potential participants failed the vision screening (one failed the VF test and the other two failed the color deficiency test) and were excluded from the study. The study was approved by the Ethics Committee of the School of Health Sciences, City, University of London (#SHS025). The research was carried out in accordance with the Declaration of Helsinki and written informed consent was obtained from all participants.

### Clinical screening for normal vision

#### Visual fields (VF)

Static threshold perimetry (24–2) was performed monocularly in both eyes using a Humphrey Field Analyser (HFA; Carl Zeiss Meditec, CA, USA) running the SITA-Standard algorithm. One participant failed due to a VF defect in one eye and was subsequently excluded from the study. For all of remaining 55 participants, the glaucoma hemifield test on the HFA was “within normal limits”.

#### Visual acuity

Recognition acuity was measured monocularly in both eyes using Early Treatment Diabetic Retinopathy Study (ETDRS) charts. All 55 participants exhibited a corrected visual acuity of 0.18 logMAR or better (Snellen equivalent of 6/9).

#### Colour deficiency

Colour vision testing was carried out binocularly using the 38-plate Ishihara pseudoisochromatic test, 2011 edition (Handaya, Tokyo, Japan,). All 55 participants correctly recognised all of the letters and patterns in the test.

### Apparatus

**Figure 5:**
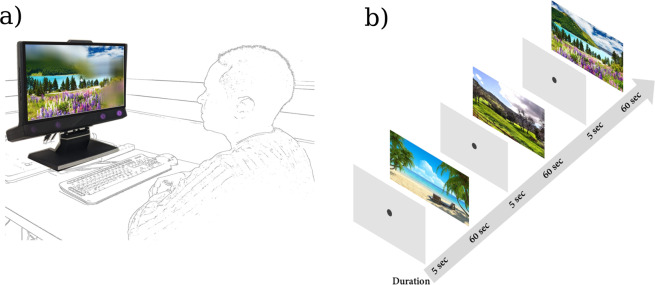
Apparatus, stimuli, and procedure. (**a**) The experiment apparatus consisted of an LCD monitor (51 × 25.5 cm; 1,920 × 1,080 pixels; 60 Hz) with an integrated eye tracker. Participants viewed the screen binocularly and were seated at approximately 60 cm away from the eye tracker without a head/chin rest, meaning that the screen subtended at a visual angle of 46° × 24°. The eye tracker allows for moderate head movements of up to 37 cm (width) × 17 cm (height) when operating at the distance of 65 cm. Participants were required only to look at the screen during test trials (no explicit response). A keyboard was used between trials to indicate readiness to continue. Changes in viewing distance were monitored using the eye tracker and the size of the artificial VF loss was dynamically adjusted accordingly. (**b**) The stimuli consisted of two video clips and three static images (only the images shown here), the latter of which were displayed for 60 seconds each. The two videos were presented for their full duration of 301 seconds and 307 seconds, respectively. The resolution of video and the images were presented at a resolution of 1,280 × 720 pixels and the frame rate of the two videos were 30 frames/second. All stimuli were displayed in a full-screen mode (resolution of 1,920 × 1,080 pixels). Stimulus order was randomised between participants. The first image (beach scene) was downloaded from http://genchi.info/beach-wallpaper-1920×1080 in March 2019.

The experiment apparatus (Fig. 5a) consisted of an LCD monitor (51 × 25.5 cm; 1920 × 1080 pixels; 60 Hz) and the Tobii TX300 eye tracker (Tobii, Danderyd, Sweden) configured to record eye gaze at 60 Hz. Our reasoning for using 60 Hz was partly pragmatic. Affordable eye trackers with similar sampling rate capabilities are available, and could be used for home monitoring (e.g., screening for VF loss while watching TV) if found to be effective^49^. This sampling rate was also matched to the refresh rate of the screen, and helped to minimize the computational overhead of generating the artificial scotoma (i.e., a higher sampling rate may have added addition delay or imprecision into to the overall system). However, there was a small amount of lag before any changes in gaze could be registered. The manufacturer claims that the overall latency of its eye tracker system is <10 msec. If we further factor in the refresh rate of the screen (60 Hz) and 3D rendering time, the total expected lag was approximately 35–45 msec. Studies^50^ have shown that system delays of up to 60 msec have an insignificant effect in disrupting perceptual processing. Moreover, the effect of system latency of gaze-contingent setups on large scotomas is low when compared with small central scotomas^51^. In the Supplemental Material we also report additional control data, collected *post-hoc*, in which no substantive differences in recorded eye-movements were observed when using a hardware setup with a higher refresh rate (240 Hz).

### Stimuli and study design

The stimuli consisted of two videos and three static images (see Fig. 5b for all images). video 1 was an aerial film of London (301 seconds) and video 2 was an advert showing attractions in London (373 seconds, from the London Vacation Travel Guide; Expedia, Bellevue, Washington, USA). The three images used were outdoor scenes, with two depicting landscapes and one depicting a beach. Unlike previous studies^10,11^ that involved a large number of images, the present study investigated the feasibility of using just a few numbers of images to detect VF loss.

**Figure 6:**
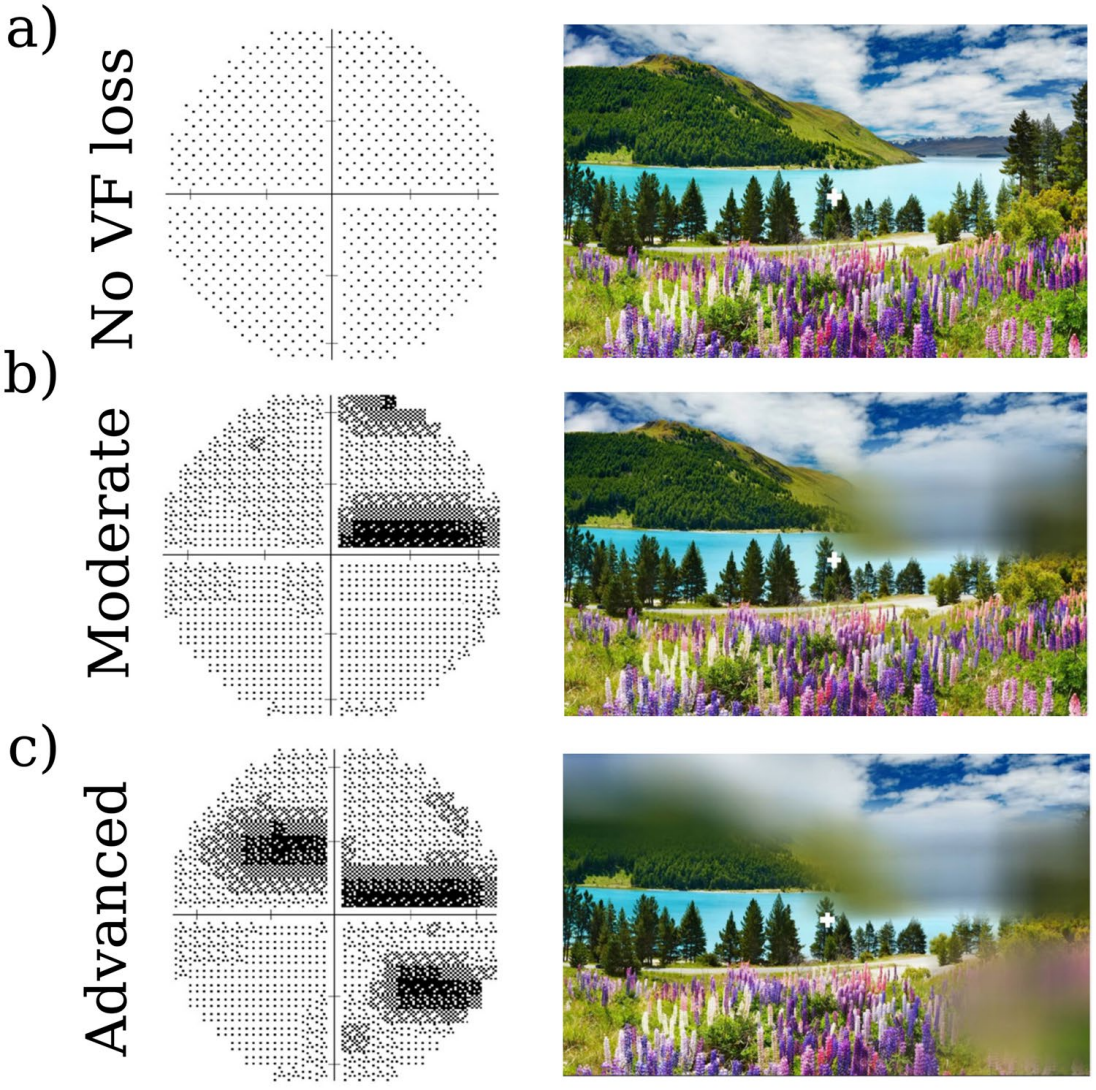
IVFs and example screenshots from the three simulated-VF loss conditions: (**a**) no VF loss. (**b**) moderate VF loss. (**c**) advanced VF loss. The impairments were constructed on monocular field data from a single real patient. The IVF was estimated by taking the maximum sensitivity from corresponding points in the left- and right-eye VFs. Note that the simulated VF loss moved depending upon the observer’s current point of gaze (white cross), and so remainined approximately static in retinal coordinates.

Custom software (GazeSS) described by Glen *et al*.^33^ was used to simulate gaze-contingent VF loss, mimicking the expected binocular VF loss based on (monocular) perimetric data from a real patient. In our implementation, blur was used since patients with early or intermediate loss often perceive VF loss as a region of a blur^42,43^. The current study used two integrated visual fields (IVFs) generated from a real glaucomatous patient’s monocular VF assessments carried out twice during the course of the condition (Fig. 6). The patient was arbitrarily selected from our database of patients with a clinically established diagnosis of open-angle glaucoma. Glaucoma was defined as VF defects in both eyes (binocular defect) with corresponding damage to the optic nerve head and an open iridocorneal drainage angle on gonioscopy. Here we simulated superior binocular visual field loss, which is representative of clinical population of patients with binocular visual field loss^52^.

The IVF estimates the binocular VF from the two monocular (left and right eye) VFs in such a way that sensitivity values for each point in the IVF were computed by taking the maximum sensitivity from corresponding points in the left and right eye VFs^35,48^. Based on the VF of the worse eye, the results of the two visits were classified as moderate (−12 ≤ MD ≤ −6 dB) and advanced VF loss (MD < −12 dB)^53^.

### Procedure

The first 45 participants were assigned randomly using a lottery to one of the three groups (i.e., no VF loss, moderate VF loss, and advanced VF loss). An additional 10 participants were subsequently recruited to the no VF loss group. These additional 10 participants were recruited as part of a related project, and were included in the present work to maximise statistical power (a posthoc power calculation^54^, considering an Cohen’s effect size of 0.5, indicated that these additional 10 controls increased statistical power by ~7%). However, the present results were qualitatively unchanged if these additional 10 observers were not included. Regardless of impairment type, all the participants watched the same set of five stimuli (i.e., video 1, video 2, and images), presented in a random order that varied among the participants. The static images were presented for 60 seconds each, and the two videos were presented for their full duration (301 seconds and 307 seconds). Prior to the presentation of each stimulus, participants were asked to fixate on a target presented in the centre of the screen. However, during the stimulus presentation interval, participants were free to move their eyes, and were encouraged to take short breaks between trials as required. Participants completed the study in a single session lasting approximately 35 minutes.

Before testing, a five-point eye tracker calibration was carried out, using the procedure provided by the manufacturer. The results of calibration were represented in a schematic representation, marking calibration points that were successfully calibrated with error lines (i.e., the differences between the position-of-gaze calculated by the eye tracker and the actual location of the calibration point) and leaving the calibration blank if there was no reading. The accuracy of the calibration was assessed by manual inspection and was repeated until “good” level was a precision was obtained (i.e., when error lines were within the circle drawn by the system).

As part of the consent procedure, participants were given an information sheet explaining the general objective of the study, which was to understand the effects of sight loss on eye movements while watching videos and images on a computer. During the study they were instructed simply to relax and look at the screen normally, as though they were watching television at home.

### Eye movement analysis

#### Preprocessing

Eye gaze was recorded binocularly at 60 Hz. The eye tracker produces raw gaze position (horizontal and vertical positioning in pixels) and distance of the participant from the tracker (in mm). All of the raw gaze data were converted to degrees visual angle using the distance information for each raw gaze positions. Saccade and fixation identification routines were adapted from PyGaze^55^. Movements were classified as saccadic if both: the velocity >30°/s, and the acceleration >8000°/s^2^. To exclude microsaccades or minor artefacts of the eye tracker, similarly to in previous studies^56^, small saccades of amplitude <0.5° were discarded post-hoc. The rest of the gaze points are considered as fixations. As recommended previously by Hoppe *et al*.^57^, any remaining fixations >500 msec were discarded^.^ This was done to remove any smooth pursuit movements. However, the results of the present study were qualitatively unchanged if this preprocessing was not performed.

Eye movement data were analysed using custom software written in MATLAB R2017a (MathWorks Inc., Natick, MA, USA). We examined four eye movement features as described below.

#### Saccade amplitude

**Figure 7:**
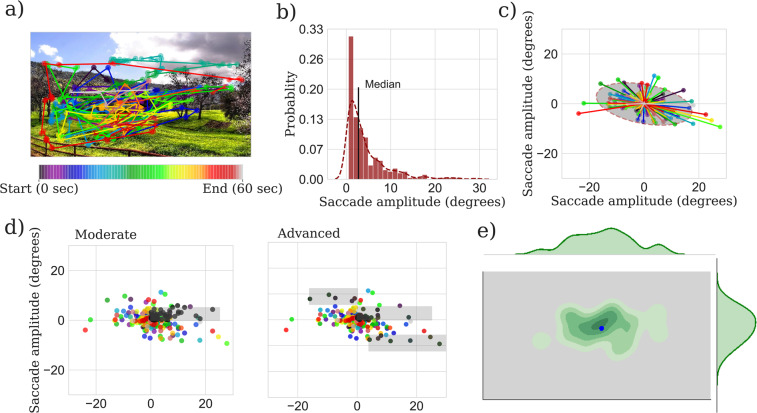
Four methods of eye movement analysis. (**a**) Example eye movements obtained from a single participant, during free-viewing of one of the static images. The colour of the scan path codes time. (**b**) Histogram of the saccades, computed from (model was used to compute the probability). The median amplitude was used as the summary measure. (**c**) Aligned plots of the saccades in the VF. The BCEA is the area of the ellipse (grey shaded region). (**d**) Saccadic movements in the direction of/landed on the pre-saccade VF loss location (black dots falling in the grey shaded region) for the moderate and advanced group. (**e**) An illustration of KDE analysis of gaze data for the frames of the videos. The KDE model was used to compute the probability score of a test point (average gaze position in a frame for a given participant, shown in blue dot).

Saccadic amplitude describes the magnitude (in degrees) of the rapid eye movements that met the ‘saccadic’ criteria (see data preprocessing) (Fig. 7a). Since the distribution of saccadic amplitudes is often non-Gaussian, we considered median values of saccade amplitude as the summary measures for statistical analyses (Fig. 7b). One median saccade amplitude value was computed per participant, per stimulus.

#### Bivariate contour ellipse area (BCEA)

BCEA was computed to measure the spread of saccadic endpoints. Following a previously described analysis approach^11^, saccade endpoints were first mapped into a location in a new plane (Fig. 7c) by first translating the saccade start position to the centre (0, 0) and then by applying the same translation to saccade endpoints. The BCEA was computed with a probability area of 95%. This produced one BCEA value per participant per stimulus.

#### Proportion of saccades landing on the pre-VF loss (SLV) location

SLV was computed as the proportion of saccades (aligned) landing on the VF loss locations to the total number of saccades (Fig. 7d). For each participant in the VF loss groups (moderate and advanced), one SLV value was computed per stimulus, while for each participant in the no VF loss group, two separate SLV values were computed (based on the scotoma locations of the moderate and advanced scotoma groups) for comparison with the moderate and advanced VF loss groups.

#### Kernel density estimate (KDE) of gaze fixations

The 3 metrics described above (4.6.3–4.6.4) have been used extensively to characterise eye movement data in the past^58,59^, and it is possible that these ‘simple’ metrics are sufficient for indexing VF loss. However, an alternative and in some ways, a more direct way of classifying an observer as healthy or abnormal is to quantify the likelihood that a given set of eye movements belongs to somebody with/without VF loss.

To do this, we developed the following ‘KDE’ procedure. In this procedure, gaze positions belonging to saccades were excluded, meaning that only available gaze positions of fixations and pursuits were considered. For each frame in every clip, the average gaze positions {(x_i_,y_i_), i = 1,…,n} of the no VF loss group participants were used to compute a model (probability mass function) based on KDE. The two gaze positions available per frames were averaged (based on 60 Hz eye tracker and 30 frames/sec video). The KDE was computed by adding Gaussians (subsequently normalised to unity), each centred at one fixation location of the participants in the no VF loss group (Fig. 7e).

1 $${\rm{f}}({\rm{x}},{\rm{y}})=\frac{1}{n}\mathop{\sum }\limits_{i=0}^{n}{K}_{{h}_{x}}({\rm{x}}\,-\,{{\rm{x}}}_{i}){K}_{{h}_{y}}({\rm{y}}\,-\,{{\rm{y}}}_{i})\text{,}$$

where ${K}_{{h}_{x}}\,{\rm{a}}{\rm{n}}{\rm{d}}\,{K}_{{h}_{y}}\,$are kernel functions: in our case Gaussian kernel, given by:

$${K}_{{h}_{x}}({\rm{x}}-{{\rm{x}}}_{i})=\frac{1}{\sqrt{2\varPi }h}\exp \left(-\frac{{({\rm{x}}-{{\rm{x}}}_{i})}^{2}}{2h}\right)\,and\,{K}_{{h}_{y}}({\rm{y}}\,-\,{y}_{i})=\frac{1}{\sqrt{2\varPi }h}\exp \left(-\frac{{({\rm{y}}-{{\rm{y}}}_{i})}^{2}}{2h}\right),$$

where the subscript *h* is the standard deviation of the Gaussian kernel.

A Gaussian kernel function with a standard deviation of h = 1.5° was chosen based on the assumption that each gaze represents a foveal vision from a participant. Fixations in proximity sum up to a higher value, whereas fixations at a distant space contribute to the density function to a small degree. A similar method has been proposed previously to compute the coincidence between the gaze points of multiple subjects^60,61^.

Before computing the KDE, outliers at each frame from the available gaze data of the no VF loss group were excluded using isolation forest method^62^. For each video and every frame, the KDE function (f(x, y)) was used to compute the probability of the average gaze positions of participants in the VF loss group. To compute the probability of gaze among participants in the no VF loss group, we used a standard “leave-one-out” method^63^. This produced one probability value for every frame of each video, for every participant (see the Fig. 1. in *Results* section for illustration). This magnitude of the probability value explains how far the spatial distance of a given participant’s gaze in a given frame is from the gaze positions of participants in the no VF loss group. The probability of a gaze point from a test participant would be higher if located closer to the gaze positions of participants in the no VF loss group. For statistical analysis, the mean of the probability scores of all frames in a video was computed (single value per participant per video). The KDE analysis was performed using the Scikit-learn package in Python^64^.

### Statistical analyses

We applied non-parametric statistical tests due to our small sample size. Kruskal–Wallis one-way analysis of variance was used to test whether there was a statistically significant difference between the three VF groups. Whenever a significant difference was found, post-hoc analyses were conducted using a Mann–Whitney U test (two-tailed) with Bonferroni corrections for multiple comparisons. The alpha level was set to 0.05.

### Further analysis to Improve separation performance

To assess the ability of individual eye movements parameters to separate between VF loss groups (no vs. moderate VF loss; no vs. advanced VF loss), receiver operating characteristics (ROCs) were computed using individual eye movement parameters (e.g., saccade amplitude, BCEA, and KDE). To try to improve the separation between the groups, multiple eye movement features were extracted and analyzed using machine learning techniques. Eighty-four additional features were extracted from the saccadic movements (see Figure S2), following similar procedure introduced by Crabb *et al*.^24^. In total, 87 features were analyzed for each video (86 features for the images). The features were serialized into a vector and were transformed into a new space using kernel principal component analysis (KPCA). The KPCA analysis extracted principal components (or features) from a high-dimensional feature space that are nonlinearly related to the input variables (i.e., features from each participant). The feature space, also known as the kernel matrix, contains distances between the features of each participant and was of size (N X N), where N is the number of participants. The distance K_ij_ between feature vectors of two participants, i and j is computed using the following Gaussian kernel:

2 $${K}_{ij}=\gamma {|{X}_{i}-{X}_{j}|}^{2}\text{.}$$

Where $\gamma$ is the variance and its value was determined using cross-validation (see Table S2). The resulting kernel matrix was normalized and decomposed to its principal components (eigenvector). The dimension of the feature vector was reduced by selecting eigenvectors with the corresponding highest eigenvalues. The resulting features were classified using AdaBoost classifier^65,66^. The AdaBoost algorithm recursively generates classifier that learns from the previous weak classifier by focusing on the hard to classify instances. The final prediction was the weighted sum of the weak classifiers. We used a Decision tree classifier as a weak learner. Best performing parameters (variance of the gaussian in KPCA, the depth of decision trees, the learning rate) were selected using cross-validation (see Table S2).

## Supplementary information

Supplemental Material

## Data Availability

The stimuli are publicly available. The data are presented in the manuscript in detail. The code used to generate the result is available upon request.
