# Supplementary material for: Using eye movements to detect visual field loss: a pragmatic assessment using simulated scotoma
Source: Sci Rep. 2020 Jun 17;10:9782. doi: 10.1038/s41598-020-66196-2 (PMC7299979; doi:10.1038/s41598-020-66196-2)
Supplement: Supplementary file 1 — Supplemental Material [file 41598_2020_66196_MOESM1_ESM.docx]

***Supplemental Material for:* Using eye movements to detect visual field loss: a pragmatic assessment using simulated scotoma**

Daniel S. Asfaw^1^, Pete R. Jones^1^, Laura A. Edwards^1^, Nicholas D. Smith^1^**,** David P. Crabb^1*^

*^1^ Division of Optometry and Visual Science, School of Health Sciences, City, University of London, London, UK.*

* Correspondence: David P. Crabb (David.Crabb.1@city.ac.uk)

**1. Additional Figures and Statistics**

***Table S1*** The results of Rank Sum test comparisons between no VF loss and moderate VF loss, and between no VF loss and advanced VF loss, each in terms of the four primary eye-movement metrics (SA, BCEA, SLV and KDE probability scores). Statistically significant (at P <0.05) associations are marked with an asterisk and highlighted in bold.

|  |  | Saccade Amplitude  (U ,P-value, η^2^) | BCEA  (U, P-value, η^2^) | SLV  (U, P-value, η^2^) | KDE Probability  (U, P-value, η^2^) |
| --- | --- | --- | --- | --- | --- |
| No VF loss  Vs  Moderate | video **1** | (144,0.33,0.03) | (163, 0.75, 0.01) | (81, 0.002, 0.22) | (72,0.002,0.26) |
|  | video **2** | (112, 0.05, 0.11) | (103,0.03, 0.14) | (52,<0.001, 0.36) | (91, 0.01,0.18) |
|  | images | **(140, 0.28, 0.04*)** | **(173,0.99,0.004*)** | **(175,0.37,0.003*)** | **N/A** |
| No VF loss  Vs  Advanced | video **1** | (79,0.003, 0.23) | (94,0.014, 0.17) | (141, 0.1, 0.04) | (111,0.05,0.11) |
|  | video **2** | (76, 0.003, 0.24) | (100,0.02, 0.15) | (130, 0.06, 0.07) | (120,0.09,0.089) |
|  | images | (68,0.001, 0.28) | **(143, 0.33,0.04*)** | (145,0.12, 0.04) | **N/A** |

***Table S2*** Parameters used to generate the ROC leave-one-out analysis shown in Figure 4 of the main manuscript.

|  |  | Gamma of the KPCA | Learning rate in AdaBoost | Number of estimators | Depth of decision tree |
| --- | --- | --- | --- | --- | --- |
| No VF loss  Vs  Moderate | video **1** | **0.003** | **0.1** | **25** | 3 |
|  | video **2** | **0.4** | **0.07** | **10** | 1 |
|  | images | **0.15** | **0.02** | **10** | 1 |
| No VF loss  Vs  Advanced | video **1** | **0.2** | **0.4** | **150** | 3 |
|  | video **2** | **0.1** | **0.004** | **10** | 1 |
|  | images | **1.13** | **0.94** | **100** | 3 |

**
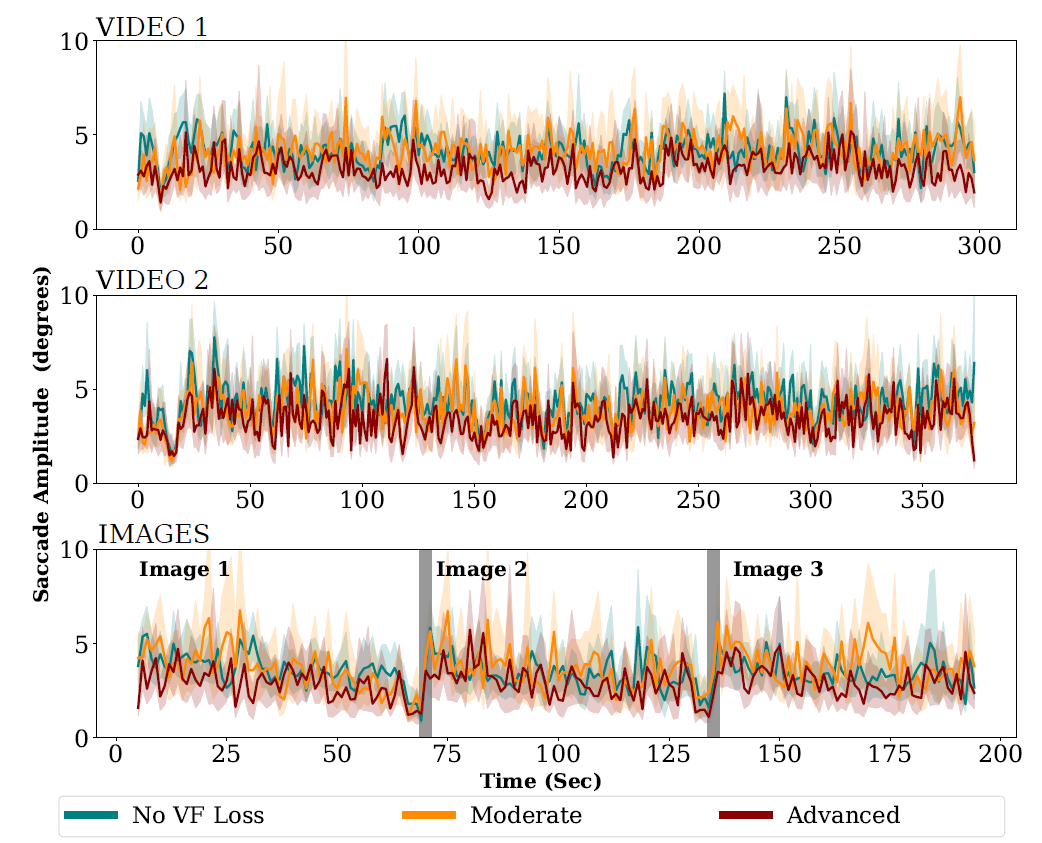
**

**Figure S1.** Saccade amplitude as a function of time for video 1, video 2, and images. For each participant, the median saccade amplitude was computed using 1-second temporal bins. The solid lines show the grand median value for each group (simulated field loss condition). The shaded regions indicate interquartile range.

**
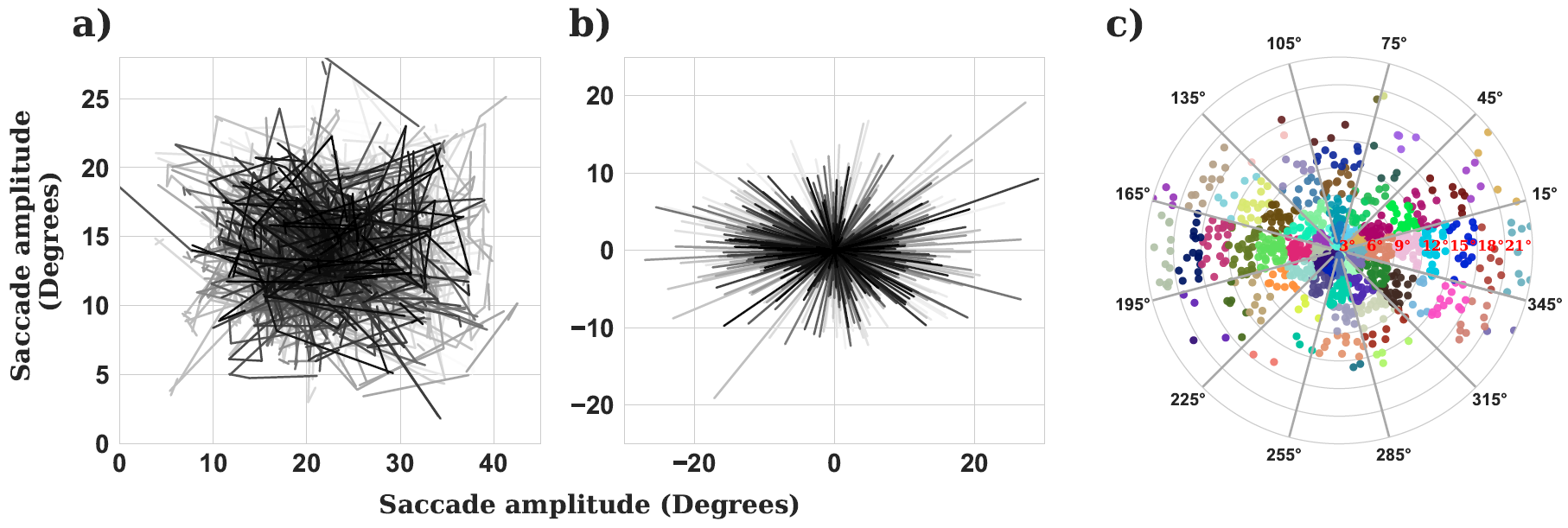
**

**Figure S2.** Method of extracting features from saccadic movements. (***a***) Example of a scanpath from a participant who watched video 2. (***b***) Each saccade is mapped to a new planned by setting their starting position to as (0,0). (***c***) Scatter plot, showing the end location of each saccade in polar coordinates. Saccades that landed in the same bin are shown using the same color. Each bin measures 3° (radial) and by 30° (angular). In total, there were 84 bins (resolution of seven in the radial direction and 12 in the angular direction). The extracted features represent the number of saccades that landed in each bin.

**2. Effect of simulating visual field loss with a higher-refresh rate monitor, and higher sampling rate eye tracking**

It is possible that the results observed in the main manuscript were affected by the refresh rate of the monitor and eye-tracker, both of which were limited to 60 hz. To investigate whether this might have been the case, we installed a new monitor with 240 Hz refresh rate (Alienware AW2518HF, Dell, Florida , USA), and reconfigured the eye tracker (Tobii TX300) to record gaze at 300 Hz.

We tested the new setup on one subject (author DA). The subject watched the same set of stimuli using both the original hardware setup, and the new setup. In each case, viewing took place under two simulated impairment conditions: “no VF loss” and “advanced VF loss”. We also used one additional video (VIDEO 3), which was not used in the main experiment due to time constraints. VIDEO 3 is an aerial film of New York, and is similar to VIDEO 2 (an aerial film of London). All stimuli were displayed in a full-screen mode (resolution of 1920 × 1080 pixels).

As in the main manuscript, the data showed that a systematic difference between the no VF loss and advanced VF loss in the two setups (see Fig S3, Fig S4). However, these differences in eye movements were similar for the two setups (saccade amplitude and BCEA reduced in advanced VF loss compared to no VF loss). This indicates that there was no substantial benefit from using a simulated scotoma with a higher temporal refresh rate.


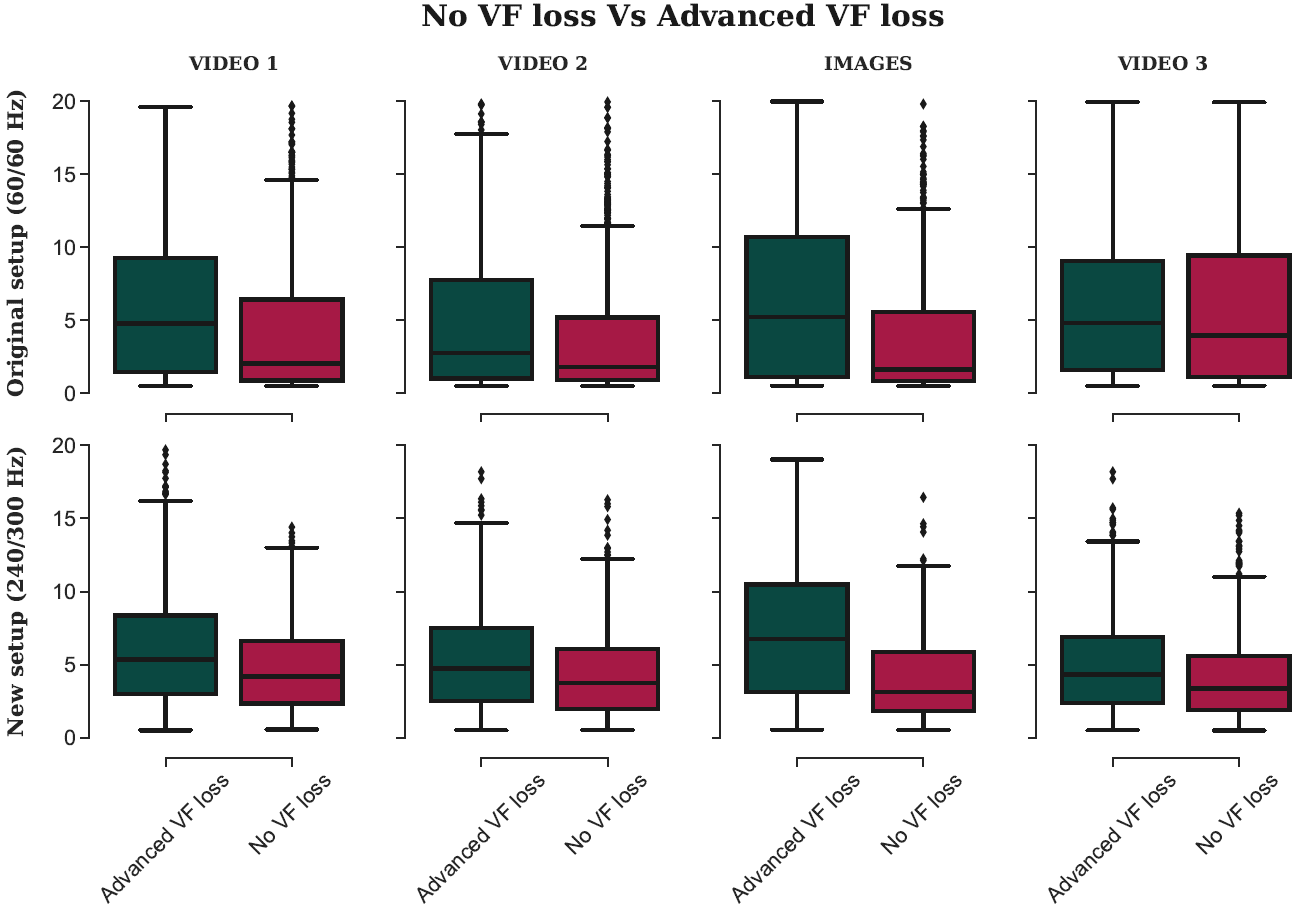


**Figure S3.** Comparison of saccade amplitude between the no VF loss and Advanced VF loss using both the original setup (top row) and new, high-frequency setup (bottom row).


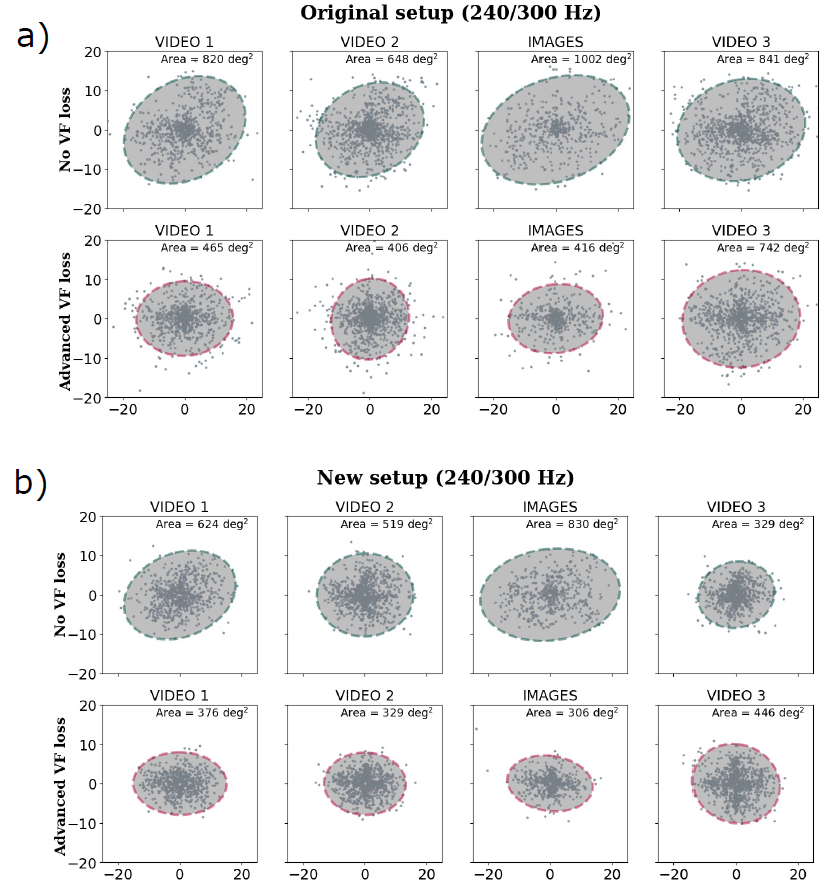


**Figure S4.** Comparison of the spread of saccade endpoints (measured using BCEA) between the No VF loss and Advanced Simulated VF loss using (a) the original setup and (b) new, high-refresh-rate setup.
